# MicroRNA-9 inhibits the proliferation and migration of malignant melanoma cells via targeting sirituin 1

**DOI:** 10.3892/etm.2021.10569

**Published:** 2021-08-06

**Authors:** Pingyuan Bu, Chengqun Luo, Quanyong He, Ping Yang, Xi Li, Dan Xu

Exp Ther Med 14:931–938, 2017; DOI: 10.3892/etm.2017.4595

Following the publication of this paper, it was drawn to the Editors’ attention by a concerned reader that the scratch-wound assay data shown in Fig. 2C were strikingly similar to data appearing in different form in other articles by different authors. Owing to the fact that the contentious data in the above article had already been published elsewhere, or were already under consideration for publication, prior to its submission to *Experimental and Therapeutic Medicine*, the Editor has decided that this paper should be retracted from the Journal. The authors were asked for an explanation to account for these concerns, but the Editorial Office did not receive a satisfactory reply. The Editor apologizes to the readership for any inconvenience caused.

